# The Lords and the Nurses

**Published:** 1892-07-09

**Authors:** 


### Passing Topics.

### THE LORDS AND THE NURSES.

The nurses have no reason to be dissatisfied with the position assigned to nursing in the Report of the Lords' Committee. Not only is there a great deal to say about them, and not a little to recommend for their benefit, but the whole tenour of the notice bestowed upon them is kindly, and in the recommendations we find little but what is designed directly to increase and promote their comfort and advantage. Nothing which concerns their well-being appears to have escaped the attention and the solicitude of the Committee, upon whose conclusions in this connection we have no criticism to offer, unless it be to suggest that their views have been almost too microscopically adjusted to allow of their being comprehensive. After all, some other things are important besides the comfort of the nursing staff, and it would have been just as well if in enforcing the need of a generous treatment of the nurses it had been made plain that the necessity might be urged with equal propriety in the interests of the patients.

Among other qualities, health, contentment, and cheerfulness are almost indispensable in a nurse, and these can only be obtained by securing for her good and sufficient dietary, adequate rest, and sanitary surroundings. We rejoice, therefore, that the influence of the Lords' Committee has been exerted on the nurses' behalf, and the more because the Committee have wisely refrained from an attempt to emphasise their recommendation by a condemnation of the great institution which has suffered from attacks so pertinaciously, and one may almost say, unscrupulously delivered, in the interest ostensibly of the nursing staff.

We must not be supposed, however, to give full assent to the recommendations in every particular. For instance, we question very much whether, situated as hospitals are, it is desirable, or even possible, to limit a nurse's working day to eight hours, and as the Lords have placed upon record that there is a "great preponderance of opinion that the health of nurses in London is good," it would appear that a reform so serious and expensive as this would be is scarcely called for in the circumstances.

There can be no doubt as to the wisdom of the suggestion that provision by way of pension should be made for deserving nurses. Nothing could better induce a feeling of communion with the institution than a knowledge that it has recognised the duty of providing for those who have served it faithfully whenever the time comes that they must cease to work. It is only what might have been expected that in making their recommendations the Lords' Committee should draw attention to the facilities offered by the existence of the Royal National Pension Fund for Nurses, the noble offspring of a noble resolve to secure for our "ministering women" a measure of comfort and independence in their old age. The report gives ample evidence of the Committee's desire to do full justice to a class which deserves well of the community, and we cannot doubt that public opinion will sustain the managers of our hospitals in any effort they may make to give effect, as far as practicable to the several recommendations the Committee have formulated.

Agricola.

